# Artificial intelligence in acute hydrocephalus: Narrowing the diagnostic gap

**DOI:** 10.1007/s00330-026-12539-y

**Published:** 2026-04-16

**Authors:** Zeynep Bendella, Habib Bendella

**Affiliations:** 1 Department of Neuroradiology, University Hospital Bonn, Bonn, Germany; 2 Clinical Neuroimaging Group, German Center for Neurodegenerative Diseases (DZNE), Bonn, Germany; 3 Center for Spine Surgery, Spine Traumatology and Neuromodulation, GFO Kliniken Rhein-Berg, Bergisch Gladbach, Germany; 4 Department of Medicine, Faculty of Health, Witten/Herdecke University, Witten, Germany

Obstructive hydrocephalus is characterized by an obstruction of cerebrospinal fluid (CSF) flow. This may rapidly lead to a critical elevation of intracranial pressure (ICP) and constitute an acute life-threatening condition. Without timely and adequate treatment, impaired consciousness, secondary brain injury, and cerebral herniation may occur as potential sequelae incompatible with life [1]. To improve patient outcomes, rapid imaging is therefore required to enable timely neurosurgical treatment, such as placement of an external ventricular drain or causal treatment of the underlying obstruction [2]. To date, cranial CT (CCT) is considered the “gold standard” for the initial assessment of obstructive hydrocephalus in patients with acute neurological worsening [3]. In routine clinical practice, emergency CCT examinations are frequently performed and interpreted under conditions of high workload and substantial time pressure [4]. For other time-critical neuroradiological conditions, delays in image interpretation may directly affect clinical decision-making and subsequent patient management [5]. To avoid such “second hits,” which may substantially impact patient outcomes, timely and reliable image interpretation is essential.

In this context, artificial intelligence (AI) has increasingly been integrated into acute neuroradiological workflows in recent years. AI-based triage systems have been developed for selected time-critical intracranial pathologies, including intracranial hemorrhage, mass effect, and large vessel occlusion, with the aim of prioritizing pathological findings for further clinical evaluation [6, 7]. So far, obstructive hydrocephalus has rarely been examined as a distinct target entity, as most available approaches focus on ventricular segmentation or composite outcome measures. As a result, these approaches do not necessarily reflect the clinically decisive aspect of acute CSF obstruction [8, 9]. At present, reliable identification of obstructive hydrocephalus in routine clinical practice remains challenging, particularly in the presence of concomitant intracerebral pathologies that may obscure its radiological presentation. Moreover, methodological studies have demonstrated that learning-based models may rely on confounding image features or indirect surrogate markers, potentially limiting robustness and clinical generalizability [8, 9].

The work presented by Ghatak et al [10] addresses a clinically relevant gap by applying a methodologically consistent external validation approach. The diagnostic performance of an AI-based model for the detection of obstructive hydrocephalus was assessed in a multicenter cohort. In total, 200 non-contrast CCT examinations were included. The reference standard was defined by an independent consensus assessment performed by experienced neuroradiologists. By extending beyond internal validation, the study aims to approximate conditions encountered in routine clinical practice. The reported results demonstrate a high diagnostic performance, with area under the curve (AUC) values approaching 0.99 for both thin and thicker slice reconstructions. Importantly, the main relevance of these findings does not lie in peak performance metrics alone. Rather, it lies in the stability of diagnostic accuracy across clinically relevant subgroups. Diagnostic performance remained largely unaffected by intracranial hemorrhage, parenchymal alterations, pre-existing ventricular drains, patient age, or scanner manufacturer. This consistent performance under potentially confounding conditions suggests that the model captures obstructive hydrocephalus as a distinct pathophysiological entity. It does not primarily rely on indirect associated findings or technical surrogate markers. In this context, the study contributes to the ongoing discussion on robustness and clinical generalizability of AI-based triage systems in acute neuroradiology.

The high negative predictive value further suggests that such a system may be particularly useful for the reliable prioritization of unremarkable examinations, especially in high-workload settings such as emergency departments or during night and weekend shifts. At the same time, analysis of misclassified cases indicates that obstructive hydrocephalus does not represent a strictly binary entity. Borderline cases with interindividual disagreement among readers reflect not only limitations of the algorithm but also the inherent diagnostic uncertainty of routine clinical practice. Such cases remain challenging even for experienced radiologists and may therefore represent a realistic target for decision support rather than automated classification.

As acknowledged by the authors, this study has several important limitations. Its retrospective design and evaluation outside a real-time clinical workflow limit immediate translation into routine practice and preclude assessment of effects on time to interpretation, clinical decision-making, or patient outcomes. In addition, consistent with current regulatory requirements for computer-assisted triage devices, the model does not provide visual explanations or localization, making it less clear why individual cases are classified as positive or negative. This observation highlights that triage systems may alter diagnostic thresholds, potentially reducing missed findings while at the same time increasing downstream clinical actions. In patients with suspected obstructive hydrocephalus, this effect is partly mitigated by the fact that imaging findings are routinely interpreted in conjunction with the clinical presentation. It will therefore be critical to consider how the model is implemented, including seamless integration into existing radiology workflows and adequate radiologist training. Finally, the model is limited to radiological pattern recognition and does not explicitly address underlying etiology; however, stable performance across older age groups suggests that age-related ventricular enlargement was not a major source of false positive classifications.

Overall, the available data suggest that AI-based systems may support the care of patients with obstructive hydrocephalus. Further evaluation in prospective studies conducted within the clinical workflow is warranted. Such studies should include patient-relevant endpoints, such as time to intervention, neurological outcome, or mortality. Potential clinical benefit will largely depend on effective integration into existing clinical workflows, adequate user training, and appropriate adjustment of decision thresholds to disease prevalence rates and care settings. AI-based triage systems should be regarded as decision-support tools rather than autonomous decision-makers and should complement clinical expertise. Continuous performance monitoring after deployment will be essential to ensure sustained reliability and to detect potential performance drift. Only under these conditions can the future role of AI-supported triage systems in acute neuroradiological care be fully realized.
